# Application of the FLP/FRT system for conditional gene deletion in yeast *Saccharomyces cerevisiae*†

**DOI:** 10.1002/yea.1895

**Published:** 2011-08-07

**Authors:** Yang-Nim Park, Daniel Masison, Evan Eisenberg, Lois E Greene

**Affiliations:** 1 Laboratory of Cell BiologyNHLBI, NIH, Bethesda, MD 20892, USA; 2 Laboratory of Biochemistry and GeneticsNIDDK, NIH, Bethesda, MD 20892, USA

**Keywords:** Flp recombinase, FRT, conditional gene deletion, yeast

## Abstract

The yeast *Saccharomyces cerevisiae* has proved to be an excellent model organism to study the function of proteins. One of the many advantages of yeast is the many genetic tools available to manipulate gene expression, but there are still limitations. To complement the many methods used to control gene expression in yeast, we have established a conditional gene deletion system by using the FLP/FRT system on yeast vectors to conditionally delete specific yeast genes. Expression of Flp recombinase, which is under the control of the *GAL1* promoter, was induced by galactose, which in turn excised *FRT* sites flanked genes. The efficacy of this system was examined using the *FRT* site-flanked genes *HSP104, URA3* and *GFP.* The pre-excision frequency of this system, which might be caused by the basal activity of the *GAL1* promoter or by spontaneous recombination between *FRT* sites, was detected ca. 2% under the non-selecting condition. After inducing expression of Flp recombinase, the deletion efficiency achieved ca. 96% of cells in a population within 9 h. After conditional deletion of the specific gene, protein degradation and cell division then diluted out protein that was expressed from this gene prior to its excision. Most importantly, the specific protein to be deleted could be expressed under its own promoter, so that endogenous levels of protein expression were maintained prior to excision by the Flp recombinase. Therefore, this system provides a useful tool for the conditional deletion of genes in yeast. Published in 2011 by John Wiley & Sons, Ltd.

## Introduction

The yeast *S. cerevisiae* is a popular model organism for studying gene function in eukaryotes. In addition to many of the yeast proteins having structural and functional conservation to protein in higher eukaryotes, yeast are very amenable to genetic manipulation (Botstein *et al.*, 1997). Many techniques have been developed to study the functions of endogenous or exogenous genes in yeast. Among the most common methods are conventional gene deletion and regulating gene expression by using temperature-sensitive mutants or constitutive/regulatable gene expression systems.

To regulate gene expression in yeast, genes are generally placed under the well-characterized inducible or repressible (regulatable) promoters. Commonly used inducible promoters include the yeast endogenous *GAL1, GAL10, CUP1* and *MAL62. GAL1* and *GAL10* are induced in the presence of galactose (Guarente *et al.*, 1982), while *CUP1* is activated in the presence of copper (Karpova *et al.*, 2008). *MAL62* promoter is induced by maltose and repressed by glucose (Finley *et al.*, 2002). The repressible promoters, such as yeast endogenous *MET3* and *MET25* (Mumberg *et al.*, 1994), can be repressed in the presence of methionine. The yeast *PHO5* promoter is negatively regulated by inorganic phosphate (Rogers *et al.*, 1982). As heterologous repressible promoters, there are the Tet-off promoters, which were adapted from bacterial *Escherichia coli* to eukaryotic gene expression systems that are repressed in the presence of tetracycline (Gossen and Bujard, 1992; Hillen and Berens, 1994). In general, these regulatable promoters provide a fast response but they have several limitations. More or less all of them cannot be shut off completely and they have basal expression activity in non-activating or repressing conditions. In addition, the expression level by these promoters is undesirably high, which can cause abnormal physiological effects, and the range of expression level is poorly regulated by adjusting the amount of inducers or repressors (Maya *et al.*, 2008). These limitations restrict the usefulness of these systems developed to control gene expression in yeast, especially for those genes whose expression level is tightly regulated by its own promoter.

An alternative way of regulating gene expression, which avoids the restrictions of the regulatable promoters, is site-specific conditional gene deletion using the FLP/FRT recombination system. A major benefit of a conditional gene deletion system is that the gene can be expressed by its own promoter before deletion, and then after gene deletion the protein can be completely diluted out by cell division. The FLP/FRT system for gene manipulation has been intensively studied and its efficacy has been demonstrated in a wide range of organisms, including bacteria, fungi, insects, plants and animals (Cox, 1983; Golic and Lindquist, 1989; Kopke *et al.*, 2010; Lloyd and Davis, 1994; O'Gorman *et al.*, 1991). The Flp (Flippase)-mediated recombination system requires only the Flp recombinase, which originated from the yeast 2 µ plasmid (Broach *et al.*, 1982), and the Flp recombinase recognition target (FRT) sites, a 34 base pair (bp) sequence that should flank the gene of interest. When the Flp recombinase is expressed, the gene flanked site directly by *FRT* sites is then excised from the genome by homologous recombination mediated by Flp recombinase (Gronostajski and Sadowski, 1985; Zhu and Sadowski, 1995). The FLP/FRT system works in a manner similar to the Cre–LoxP recombination system of bacteriophage P1, which has also been used widely in various organisms for studying function of genes. Although the Cre–LoxP system has been applied in yeast to turn off/on gene expression in a regulated manner, or to produce gene deletion mutants (Guldener *et al.*, 1996; Sauer, 1987), a FLP/FRT recombination system has not yet been developed for the conditional gene deletion in yeast.

In this study, we have established a conditional gene deletion method by using the FLP/FRT recombination system on a set of yeast shuttle vectors. Testing the efficiency of this conditional gene deletion system, we determined that the critical pre-excision rate for this system is < 2%, while the efficiency of excision is > 96%. Fluorescence imaging of yeast cells expressing GFP from a *FRT*-flanked gene shows that the protein is excised within 9 h of Flp induction and then the GFP is diluted out by cell division. Therefore, the FLP/FRT site-specific recombination system provides a powerful tool to knock out genes in yeast conditionally.

## Materials and methods

### Strains and media

Yeast strains 779-6A (*MAT***a**, *kar1*-*1, SUQ5, ade2*-*1, his3*Δ*202, leu2*Δ*1, trp1*Δ*63, ura3*-*52)* (Jung and Masison, 2001) and W303 (*MAT***a**, *ade2*-*1*, *ura3*-*1*, *his3*-*11*, *trp1*-*1*, *leu2*-*3*, *leu2*-*112*, *can1*-*100*, *GAL, SUC*) (ATCC 208 352) were used. The 779-6A strain used throughout this study is [*PSI*^+^], since it contained the prion form of the Sup35 protein. The W303 strain is used commonly in yeast research. *HSP104* was deleted in strain 779-6A by transformation, using a PCR-amplified *Kan*MX disruption cassette from a yeast deletion strain (ATCC) (Jones *et al.*, 2004). Yeast were grown at 30 °C in SD (synthetic defined; Sunrise Science Products) medium containing 2% glucose, 7 g/l yeast nitrogen base (YNB; Sunrise Science Products) and the appropriate complete supplement mixture (CSM) to maintain plasmid(s) selection. YPD medium contains 1% yeast extract, 2% peptone and 2% dextrose. 1/2YPD is similar to YPD but contains 0.5% yeast extract. The galactose medium contains 2% galactose and 2% raffinose in place of glucose. Cultures were maintained in log phase (OD_600_ < 0.6) by periodic dilution with fresh medium. *E. coli* strain DH5α was used for plasmid propagation.

### Plasmid constructions

*HSP104* (position − 844 to + 2965) flanked by *FRT* sites was amplified from genomic DNA of *S. cerevisiae* strain 779-6A with the primer pair HSP104-1 (5′-taggatccgaagttcctattctctagaaagtataggaacttcgactg ctcttgcacagaacctccc-3′) and HSP104-2 (5′-tactcgag gaagttcctatactttctagagaataggaacttcctttagttatcaacgcc atatgtccc-3′). The PCR product was digested with *Bam*HI and *Xho*I and cloned in pRS314 to generate pC4F-HSP104. To construct pC4FURA3, *Kpn*I–*Sac*I *URA3* (from − 225 to + 63) fragment flanked by 34 bp *FRT* was amplified by PCR from the plasmid pRS316 with the primer pair URA3–7 (5′-at gagctcgaagttcctattctctagaaagtataggaacttcgggcccttttc aattcaattcatcatttttttt-3′) and URA3-8 (5′-atggtaccga agttcctatactttctagagaataggaacttccggccgtaataactgatat aattaaattga-3′), digested with *Kpn*I–*Sac*I and inserted into the plasmid pRS314. The *Apa*I–*Not*I *URA3* fragment in plasmid pC4FURA3 was replaced with *Apa*I–*Eag*I-digested multi-cloning site (MCS) from the plasmid pRS314 and this resulted in pC4FMCS. Plasmid pC4FGFP was constructed by inserting *Apa*I–*Not*I fragment from pJ543 containing the ORF of *GFP*, which is under the control of *SUP35* promoter (from − 600 to − 2), and fused to *SUP35* terminator (182 bp of 3′ UTR), into the *Apa*I–*Eag*I-digested pC4FMCS. The ORF of *FLP* was amplified from plasmid pTET23 (Park and Morschhauser, 2005) by using the primer pair FLP2 (tataggatccatgtcacaatttgatatattatgtaaaacacc) and FLP3 (tatacccgggttatatgcgtctatttatgtaggatgaaag g), then inserted into *Xho*I–*Bam*HI-digested *GAL1* promoter fragment and *Xma*I–*Sac*I terminator fragment of *HSP104* into *Xho*I–*Sac*I-digested pRS315 to create pC5GAL1–FLP. Underlined sequences in the primers indicate the inserted restriction sites. All sequences have been verified. All the backbone plasmids have been described in detail (Sikorski and Hieter, 1989).

### Determination of pre-excision rate under non-inducing conditions

To determine the [*PSI*^+^]/[*psi*^−^] phenotype, cells of the Δ*hsp104* [*PSI*^+^] strain having plasmids pC4F-HSP104 and pC5GAL1–FLP were grown in SD—Trp—Leu drop-out medium to mid-log phase and then plated onto SD—Trp—Leu drop-out agar plate containing 10 µg/ml adenine. After 5 days of incubation at room temperature, the percentage of [*psi*^−^] red colonies, which represents the pre-excision rate of *HSP104*, was determined. The determined data include some ‘spontaneous’ prion loss, whose frequency, determined by the strain having only pC4F-HSP104, was < 0.2%, could be due to increased Hsp104 in cells that have more than one copy of the plasmid.

### Measurement of *URA3* gene-flipping efficiency

Cells of strain 779-6A or W303 having plasmids pC4FURA3 and pC5GAL1–FLP grown in SD—Trp—Leu—Ura drop-out medium were transferred into similar SD-Trp-Leu medium with galactose in place of dextrose to induce Flp recombinase. During growth in galactose medium, aliquots of cells at the indicated generations (see Results), as determined by doubling of OD_600_, were plated onto both YPD and uracil drop-out agar plates. To calculate the number of cells per 1 ml culture, cells in the aliquot were counted using a cell-counting chamber (Neubauer, 0.0025 mm^2^). After 3 days of incubation at 30 °C, the colonies were counted and the excision rate was calculated as colony number on uracil drop-out agar plates vs colony number on YPD plates, or the counted cell number.

### Imaging

The GFP fluorescence in yeast was imaged using the Zeiss LSM510 confocal microscope with a × 63 objective. The same settings were used for imaging the GFP fluorescence in the different strains throughout the experiment.

## Results

### Design of conditional gene deletion by using the FLP/FRT system on yeast shuttle vectors

**Figure 1:**
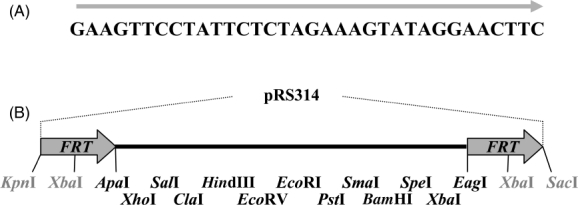
Sequence of *FRT* (A) and available restriction sites between *FRT* sites on the yeast shuttle vector (B). The restriction sites between direct repeat *FRT* sites on the plasmid pC4FMCS are in dark letters. Figures are not drawn to scale. Size of the fragment is 168 bp

In order to delete the gene of interest conditionally, this gene, along with its promoter and terminator regions, were inserted into the MCS sites flanked by the *FRT* target sequence of Flp recombinase (see Materials and methods). The 34 bp *FRT* sequence used in this study is shown in Figure 1A. The MCS flanked by *FRT* sites was inserted into centromeric plasmid pRS314, which served as the backbone for the yeast shuttle vector. The restriction sites within MCS, which are available for the insertion of the gene of interest, are shown in Figure 1B. *FLP* under control of the *GAL1* promoter on yeast centromeric shuttle vector pRS315 was used to express Flp recombinase when induced in galactose medium. These two yeast vectors for the conditional gene deletion system were transformed into either 779-6A or W303 yeast strains and were then maintained stably by growth of yeast in selection media.

### Determination of the pre-excision rate of the conditional gene deletion system

**Figure 2:**
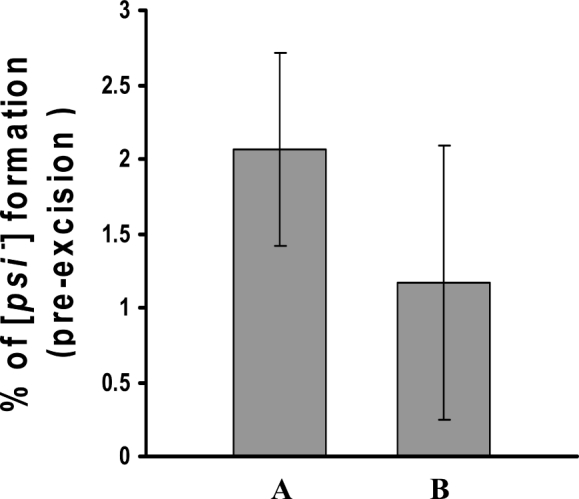
Pre-excision frequency of the conditional gene deletion system. [*PSI*^+^] cells having conditionally deletable *HSP104* were plated from the active growing culture onto agar plates containing 10 µg/ml adenine. After 5 days incubation, the percentage of [*psi*^−^] colonies was determined. The average formation of [*psi*^−^] cells from two independent transformants (A and B) was determined from six different experiments. Error bars indicate standard deviation (SD)

It is important that the conditional gene deletion system is tightly controlled, so that there is no significant excision prior to induction of Flp recombinase under non-inducing conditions. To examine the pre-excision rate of this conditional gene deletion system, we used the colony colour screening method for yeast [*PSI*^+^] and [*psi*^−^] prion phenotypes. The *ade2-1* of 779-6A strain in [*psi*^−^] cells causes adenine auxotrophy. When the growth medium contains a limited amount of adenine [*psi*^−^] cells are red, due to the accumulation of a metabolite of the adenine biosynthetic pathway. In [*PSI*^+^] cells, the Sup35 translation termination factor is in aggregated prion form, which reduces translation termination fidelity. The suppression of *ade2-1* is allowed by *SUQ5* tRNA in the [*PSI*^+^] cells, conferring adenine prototrophy, and the cells form white colonies (Wickner *et al.*, 2007). The molecular chaperone Hsp104 is necessary for replication of [*PSI*^+^] prions, so depletion of Hsp104 following conditional deletion of the *HSP104* gene cures cells of [*PSI*^+^] (Chernoff *et al.*, 1995). This curing results in a change of colony colour from white colonies ([*PSI*^+^] yeast) to red colonies ([*psi*^−^] yeast). To screen for basal pre-excision in the absence of induction of Flp by galactose, we constructed a [*PSI*^+^] strain in which genomic *HSP104* is deleted and *HSP104* flanked by *FRT* sites on plasmid pC4F-HSP104 provides Hsp104 to maintain the [*PSI*^+^] prion. This strain was then transformed by plasmid pC5GAL1–FLP, which contains *FLP* under control of the galactose-inducible *GAL1* promoter. To monitor frequency of loss of [*PSI*^+^] under non-inducing conditions, [*PSI*^+^] cells with both plasmids were grown in glucose medium to mid-log phase and then plated onto solid medium having a limiting amount of adenine. Since both [*PSI*^+^] and [*psi*^−^] colonies grow well on this culture medium, there is no selection for gene excision. In [*PSI*^+^] yeast having only pC4F-HSP104, there was no significant (<0.2%) spontaneous conversion of [*PSI*^+^] to [*psi*^−^] yeast. In two independent transformants, the frequency of formation of [*psi*^−^] cells from yeast containing deletable *HSP104* by Flp was 2.06 ± 0.65% and 1.17 ± 0.92%, respectively (Figure 2). This pre-excision frequency shows the extent of excision of *HSP104* under non-inducing conditions of Flp recombination, i.e. caused by the basal activity of the *GAL1* promoter or the spontaneous recombination between *FRT* sites. Therefore, the regulatable *GAL1* promoter is controlled relative tightly, in agreement with other published data (Flick and Johnston, 1990; Mumberg *et al.*, 1994), so there is no significant excision of the *FRT* flanked gene prior to induction of Flp recombinase.

### Efficiency of *URA3* gene deletion by Flp recombination

**Figure 3:**
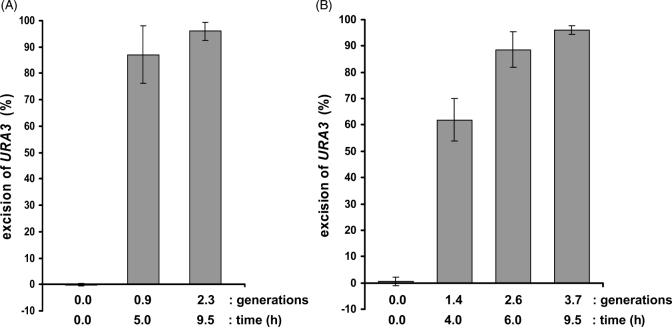
Excision efficiency of the conditional gene deletion system. (A) Yeast 779-6A and (B) W303 having plasmids pC4FURA3 and pC5GAL1-FLP were grown in SD—Trp—Leu—Ura drop-out medium prior to transfer into SD-Trp-Leu galactose drop-out medium. During growth, aliquots of cells at the indicated times (h)/generations were plated onto YPD and SD—URA plates. After incubating for 3 days at 30 °C, the excision rate was quantified as a percentage of Ura^−^ colonies. The excision values are from three independent experiments. Error bars indicate SD

In order for the FLP/FRT system to be a useful tool for engineering conditional gene deletion, the Flp recombinase must have a high efficiency of excision of the *FRT* site-flanked gene. The *URA3* gene was used to determine the efficiency of excision in both the 779-6A and W303 yeast strains. Both of these yeast strains were transformed with the plasmid pC5GAL1–FLP containing *FLP* under the control of *GAL1* promoter and the plasmid pC4FURA3, containing *URA3* flanked by *FRT* sites (see Materials and methods). The induction of Flp recombinase causes excision of *URA3* and results in uracil auxotrophy. To eliminate *ura3* cells caused by pre-excision of the *URA3* gene, cells were pre-incubated in uracil depletion medium before induction of Flp expression. After one generation (5 h growth) in galactose medium, about 85% of the 779-6A cells were uracil auxotrophs, and after two generations (9 h), ca. 96% of cells were uracil auxotrophs (Figure 3A). After three generations (>10 h), although we obtained more than 350 colonies on YPD plates, there were no colonies detected on uracil depletion medium, indicating complete excision of *URA3*. As shown in Figure 3B, cells of the strain W303 were ca. 62% uracil auxotrophs after 4 h growth, equivalent to 1.4 generations in galactose medium. After further incubation for 2.4 generations (6 h) and 3.8 generations (9 h) in glactose medium, 89% and 96% of cells resulted in uracil auxotrophs, respectively. Therefore, in both strains of yeast, this conditional gene deletion system responds rapidly with high excision efficiency and only slight heterogeneity in gene deletion from cell to cell. Interestingly, comparison of the data in Figure 3A and B suggests that the rate of gene deletion by Flp recombinase is a time-dependent, and not a generation-dependent, process. Specifically, both yeast strains show a very similar time dependency for excision, even though W303 strain grew significantly faster than 779-6A yeast strain.

### Fluorescence imaging of GFP to follow conditional gene deletion using the FLP/FRT system

**Figure 4:**
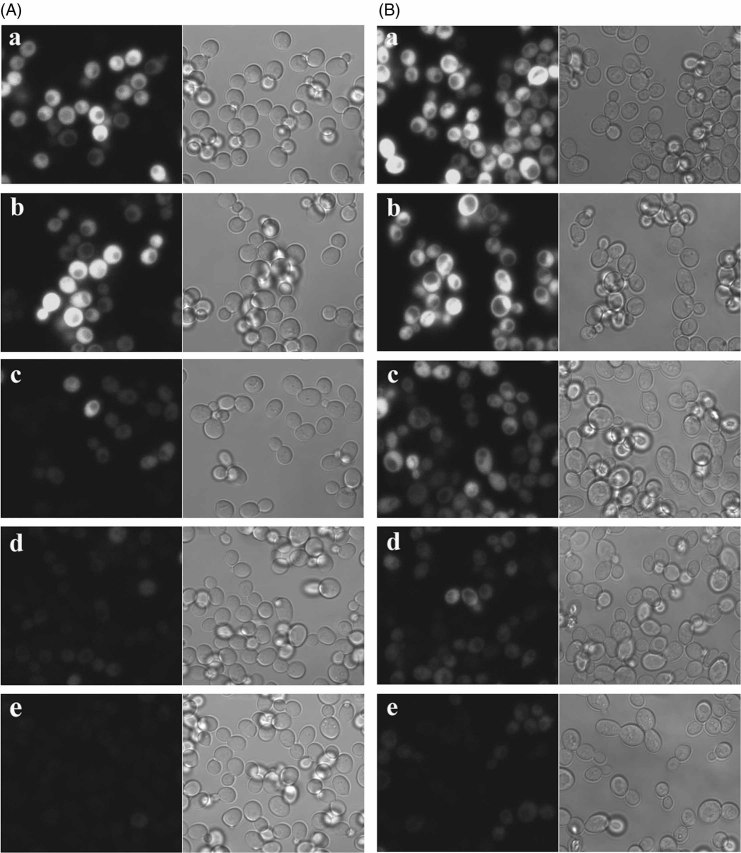
Fluorescence imaging of cells undergoing conditional deletion of *GFP* by Flp recombinase. (A) 779-6A yeast with pC4FGFP (a) were grown in glucose medium. Cells having the plasmids pC4FGFP and pC5GAL1-FLP grown in glucose medium (b) were transferred into galactose medium and incubated further for 12 (3.5 generations) (c), 17.5 (five generations) (d), and 21 (eight generations) (e) h. (B) W303 yeast with pC4FGFP (a) were grown in glucose medium. Cells having the plasmids pC4FGFP and pC5GAL1-FLP grown in glucose medium (b) were transferred into galactose medium and incubated further for 9 (four generations) (c), 14 (six generations) (d) and 18 (7.5 generations) (e) h. The cells were actively growing

To follow gene excision using the FLP/FRT recombination system in real time, we transformed 779-6A and W303 yeast strains with a *GFP* marker gene flanked by *FRT* sites under the control of the yeast *SUP35* constitutive promoter. No detectable GFP fluorescence was detected in strain 779-6A and W303 yeast strains without the GFP expression vector (data not shown), while the yeast containing only pC4FGFP having *GFP* flanked by *FRT* sites showed strong fluorescence in both glucose (Figure 4A, Ba) and galactose medium (data not shown). Moreover, the presence of pC5GAL1–FLP plasmid had no significant effect on GFP fluorescence when the cells were grown in SD medium, conditions in which there is no induction of Flp expression (Figure 4A, Bb). More than 90% of the yeast cells showed GFP fluorescence. However, there was a wide range in fluorescence intensity of the GFP, although *GFP* was on a centromeric plasmid which made it difficult to detect the fluorescence in low-expressing cells using the same settings of the microscope. Following induction of Flp expression by growing both strains in galatose medium, there was no significant change in GFP fluorescence intensity for the first 6–7 h (data not shown), but after 9 h there was slight reduction in GFP intensity, as shown in Figure 4Bc. However, after growth for 12–14 h in galactose there was a marked reduction in GFP intensity, and by 18 h in galactose medium the fluorescence was no longer detectable (Figure 4Ac–e, Bd, e). Here again, excision of the *GFP* gene appeared to occur in a time-dependent process, since the W303 strain grew faster than the 779-6A strain, but both strains showed that the *GFP* gene was excised 7–9 h after Flp induction. Following excision, the GFP fluorescence is gradually diluted out by cell division (Figure 4A, Bc–e), since degradation occurs on a much slower time scale (Grilly *et al.*, 2007). In addition, we were able to determine the percentage of yeast cells of both strains, in which the *GFP* was not excised after 18 h of induction of Flp recombination. By confocal imaging, < 0.7% of cells were found to have strong GFP fluorescence, which might be due either to loss of the plasmid pC5GAL1–FLP or lack of Flp expression. These results demonstrate that the conditional gene deletion system established in this study provides a useful tool for studying the function of both endogenous and exogenous genes in yeast.

## Discussion

In this study, a conditional gene deletion system using the yeast FLP/FRT system was successfully established to study the function of genes in yeast. This system was engineered to be easily manipulative, since the yeast shuttle vector contained a multicloning site, MCS, inserted between *FRT* sites. The gene of interest with its up- and downstream regulatory regions can readily be inserted into the MCS by a single cloning step. Furthermore, the gene flanked by *FRT* sites can be subcloned into other yeast shuttle vectors or integrated into the ectopic locus in the yeast genome by PCR-based gene integration (Wendland, 2003). The genomic integration of the *FRT* site-flanked gene cassette abolishes such problems as losing the plasmid and heterogeneous expression level due to variation in number of copies of this plasmid per cell. Since it is essential that the gene of interest is only expressed from the *FRT*-flanked locus, the large-scale yeast gene deletion library (ATCC; http://www-sequence.stanford.edu/group/yeast_deletion_project/deletions3.htm) is a valuable resource to obtain parent yeast strains as a source of alleles for chromosomal deletion. In this study, two different yeast shuttle vectors were used to express Flp and the gene of interest, but a single vector can be engineered to express both these proteins, which may be important if there are a limited number of selection marker genes.

An important feature of this conditional deletion system is that the gene of interest is under the control of its own promoter. Therefore, its regulation is not different from control cells, which in turn results in its protein level being similar to the endogenous level prior to induction of Flp recombinase. This characteristic of this system is especially beneficial for studying genes in which the endogenous gene expression level is important for function. For example, the molecular chaperone Hsp104 is necessary to maintain [*PSI*^+^] prion, therefore it can not be deleted, yet when Hsp104 is overexpressed it cures cells of [*PSI*^+^] prions (Chernoff *et al.*, 1995; Newnam *et al.*, 1999). Thus, other heterologous repressible promoters that overexpress Hsp104 under non-repressible conditions cannot be used to study Hsp104 function. Although it is often an advantage to use the endogenous promoter to study gene function, this conditional gene deletion system is not limited to using the endogenous promoter. The conditional gene deletion system can also be engineered to excise the gene of interest expressed from a heterologous promoter, if the expression level of the gene needs to be changed from the normal expression level.

This conditional deletion system can be applied to study essential gene function, as was done previously using the tetracycline-repressible promoters (Yu *et al.*, 2006). Essential gene function is often studied by making temperature-sensitive mutants. However, it is difficult to generate these temperature-sensitive mutants in a systematic manner and they are rapidly degraded at the non-permissive temperature. In addition, since temperature-sensitive mutants have a mutation in the primary sequence, this may cause secondary defects.

Another advantage of this system is that it provides relatively fast gene deletion. The deletion efficiency reached ca. 85% within 5–6 h by inducing expression of Flp. Obviously, deletion is not synchronized in a yeast population, but once the Flp recombinase is induced by galactose, > 96% of cells showed deletion of genes after growth for 9 h. As expected, the recombination efficiency is affected by the size of gene between *FRT* sites, i.e. there is a decrease in excision efficiency with increasing size of the gene flanked by the *FRT* sites (Liu *et al.*, 2002). In fact, we observed that the excision of *HSP104* was slower than that of *GFP* analysed by Western blot analysis of Hsp104 protein levels (data not shown). This indicates the possibility that the size of the *FRT* site-flanked gene affects the efficiency of excision of the gene.

Aside from all these advantages, this system has obvious disadvantages, similar to other gene regulatory systems. One disadvantage of this system is that in yeast the most tightly repressed *GAL1* promoter, which regulates the expression of Flp recombinase, is itself slightly leaky in glucose medium, as indicated by the pre-excision frequency of < 2% in glucose medium. Furthermore, to induce Flp, the growth medium has to be exchanged from glucose to galactose. To avoid this change of growth conditions, the Tet-on systems (Belli *et al.*, 1998; Urlinger *et al.*, 2000), which are turned on by adding inducer without changing the growth conditions, are another option for the regulation of Flp expression. As with the other promoters, with the Tet-on system it is important to control for pre-excision events, due to the leakiness of the promoter under non-inducing conditions. Leaky pre-excision events can be prevented in many ways. For example, in the case of conditional deletion of the *URA3* gene, growth in uracil depletion medium before inducing Flp recombinase can be used to select against such events. When this system is used for studying the function of essential genes, there is automatic selection for pre-excision events because cells having such an event will not survive. Another obvious disadvantage of the conditional deletion system is that the deletion of the gene is permanent, therefore expression from this gene is irreversible. To introduce reversible gene expression, a second inducible promoter has to be considered, but again this introduces the problem of having a tightly controlled promoter for the yeast system. Finally, the protein that was expressed from the gene of interest prior to its excision is reduced by a combination of degradation and cell division. Yeast proteins have been shown to have a wide range of half-lives (Belle *et al.*, 2006), so that a protein with a long-half-life requires many cell divisions to dilute out the protein. Given these limitations, this FLP/FRT system offers an easy way to study the biological function of proteins in yeast by conditional gene deletion.

## References

[b1] Belle A, Tanay A, Bitincka L (2006). Quantification of protein half-lives in the budding yeast proteome. Proc Natl Acad Sci USA.

[b2] Belli G, Gari E, Piedrafita L (1998). An activator/repressor dual system allows tight tetracycline-regulated gene expression in budding yeast. Nucleic Acids Res.

[b3] Botstein D, Chervitz SA, Cherry JM (1997). Yeast as a model organism. Science.

[b4] Broach JR, Guarascio VR, Jayaram M (1982). Recombination within the yeast plasmid 2 µ circle is site-specific. Cell.

[b5] Chernoff YO, Lindquist SL, Ono B, W (1995). Role of the chaperone protein Hsp104 in propagation of the yeast prion-like factor [psi^+^]. Science.

[b6] Cox MM (1983). The FLP protein of the yeast 2 µ plasmid: expression of a eukaryotic genetic recombination system in *Escherichia coli*. Proc Natl Acad Sci USA.

[b7] Finley RL, Zhang H, Zhong J, Stanyon CA (2002). Regulated expression of proteins in yeast using the MAL61–62 promoter and a mating scheme to increase dynamic range. Gene.

[b8] Flick JS, Johnston M (1990). Two systems of glucose repression of the GAL1 promoter in *Saccharomyces cerevisiae*. Mol Cell Biol.

[b9] Golic KG, Lindquist S (1989). The FLP recombinase of yeast catalyzes site-specific recombination in the *Drosophila* genome. Cell.

[b10] Gossen M, Bujard H (1992). Tight control of gene expression in mammalian cells by tetracycline-responsive promoters. Proc Natl Acad Sci USA.

[b11] Grilly C, Stricker J, Pang WL (2007). A synthetic gene network for tuning protein degradation in *Saccharomyces cerevisiae*. Mol Syst Biol.

[b12] Gronostajski RM, Sadowski PD (1985). The FLP recombinase of the *Saccharomyces cerevisiae* 2 µ plasmid attaches covalently to DNA via a phosphotyrosyl linkage. Mol Cell Biol.

[b13] Guarente L, Yocum RR, Gifford P (1982). A GAL10–CYC1 hybrid yeast promoter identifies the *GAL4* regulatory region as an upstream site. Proc Natl Acad Sci USA.

[b14] Guldener U, Heck S, Fielder T (1996). A new efficient gene disruption cassette for repeated use in budding yeast. Nucleic Acids Res.

[b15] Hillen W, Berens C (1994). Mechanisms underlying expression of Tn10 encoded tetracycline resistance. Annu Rev Microbiol.

[b16] Jones G, Song Y, Chung S, Masison DC (2004). Propagation of *Saccharomyces cerevisiae* [PSI^+^] prion is impaired by factors that regulate Hsp70 substrate binding. Mol Cell Biol.

[b17] Jung G, Masison DC (2001). Guanidine hydrochloride inhibits Hsp104 activity *in vivo*: a possible explanation for its effect in curing yeast prions. Curr Microbiol.

[b18] Karpova TS, Kim MJ, Spriet C (2008). Concurrent fast and slow cycling of a transcriptional activator at an endogenous promoter. Science.

[b19] Kopke K, Hoff B, Kuck U (2010). Application of the *Saccharomyces cerevisiae* FLP/FRT recombination system in filamentous fungi for marker recycling and construction of knockout strains devoid of heterologous genes. Appl Environ Microbiol.

[b20] Liu P, Jenkins NA, Copeland NG (2002). Efficient Cre–loxP-induced mitotic recombination in mouse embryonic stem cells. Nat Genet.

[b21] Lloyd AM, Davis RW (1994). Functional expression of the yeast FLP/FRT site-specific recombination system in *Nicotiana tabacum*. Mol Gen Genet.

[b22] Maya D, Quintero MJ, de la Cruz Munoz-Centeno M, Chavez S (2008). Systems for applied gene control in *Saccharomyces cerevisiae*. Biotechnol Lett.

[b23] Mumberg D, Muller R, Funk M (1994). Regulatable promoters of *Saccharomyces cerevisiae*: comparison of transcriptional activity and their use for heterologous expression. Nucleic Acids Res.

[b24] Newnam GP, Wegrzyn RD, Lindquist SL, Chernoff YO (1999). Antagonistic interactions between yeast chaperones Hsp104 and Hsp70 in prion curing. Mol Cell Biol.

[b25] O'Gorman S, Fox DT, Wahl GM (1991). Recombinase-mediated gene activation and site-specific integration in mammalian cells. Science.

[b26] Park YN, Morschhauser J (2005). Tetracycline-inducible gene expression and gene deletion in *Candida albicans*. Eukaryot Cell.

[b27] Rogers DT, Lemire JM, Bostian KA (1982). Acid phosphatase polypeptides in *Saccharomyces cerevisiae* are encoded by a differentially regulated multigene family. Proc Natl Acad Sci USA.

[b28] Sauer B (1987). Functional expression of the cre–lox site-specific recombination system in the yeast *Saccharomyces cerevisiae*. Mol Cell Biol.

[b29] Sikorski RS, Hieter P (1989). A system of shuttle vectors and yeast host strains designed for efficient manipulation of DNA in *Saccharomyces cerevisiae*. Genetics.

[b30] Urlinger S, Baron U, Thellmann M (2000). Exploring the sequence space for tetracycline-dependent transcriptional activators: novel mutations yield expanded range and sensitivity. Proc Natl Acad Sci USA.

[b31] Wendland J (2003). PCR-based methods facilitate targeted gene manipulations and cloning procedures. Curr Genet.

[b32] Wickner RB, Edskes HK, Shewmaker F, Nakayashiki T (2007). Prions of fungi: inherited structures and biological roles. Nat Rev Microbiol.

[b33] Yu L, Pena Castillo L, Mnaimneh S (2006). A survey of essential gene function in the yeast cell division cycle. Mol Biol Cell.

[b34] Zhu XD, Sadowski PD (1995). Cleavage-dependent ligation by the FLP recombinase. Characterization of a mutant FLP protein with an alteration in a catalytic amino acid. J Biol Chem.

